# Predictability of the impact of multiple stressors on the keystone species *Daphnia*

**DOI:** 10.1038/s41598-018-35861-y

**Published:** 2018-12-04

**Authors:** Maria Cuenca Cambronero, Hollie Marshall, Luc De Meester, Thomas Alexander Davidson, Andrew P. Beckerman, Luisa Orsini

**Affiliations:** 1 Environmental Genomics Group, School of Biosciences, the University of Birmingham, Birmingham, B15 2TT UK; 2 Laboratory of Aquatic Ecology, Evolution and Conservation, University of Leuven, Ch. Deberiotstraat 32, 3000 Leuven, Belgium; 3 Lake Group, Department of Bioscience, Aarhus University, Vejlsøvej 25, P.O. Box 314, DK-8600 Silkeborg, Denmark; 4 Department of Animal and Plant Sciences, University of Sheffield, Sheffield, S10 2TN UK

## Abstract

Eutrophication and climate change are two of the most pressing environmental issues affecting up to 50% of aquatic ecosystems worldwide. Mitigation strategies to reduce the impact of environmental change are complicated by inherent difficulties of predicting the long-term impact of multiple stressors on natural populations. Here, we investigated the impact of temperature, food levels and carbamate insecticides, in isolation and in combination, on current and historical populations of the freshwater grazer *Daphnia*. We used common garden and competition experiments on historical and modern populations of *D. magna* ‘resurrected’ from a lake with known history of anthropogenic eutrophication and documented increase in ambient temperature over time. We found that these populations response dramatically differed between single and multiple stressors. Whereas warming alone induced similar responses among populations, warming combined with insecticides or food limitation resulted in significantly lower fitness in the population historically exposed to pesticides. These results suggest that the negative effect of historical pesticide exposure is magnified in the presence of warming, supporting the hypothesis of synergism between chemical pollution and other stressors.

## Introduction

Anthropogenic stressors have been responsible for an average population decline in all ecosystems, with the most severe impact recorded in freshwater ecosystems, where 76% of species have experience a decline since1970^1–4^. In particular, human-driven hyper-eutrophication and global warming have affected up to 50% of freshwater ecosystems worldwide in the last century^5,6^.

Mitigation strategies to reduce the impact of environmental change are limited because the effect of multiple stressors can be synergistic (greater than the sum of individual effects), antagonistic (resulting from the contrasting action of two or more stressors) or additive (sum of the individual effects)^7^, making predictions of the impact of multiple stressors compared to a single stressor challenging^8,9^. Furthermore, population level responses can vary dramatically because of differences in environmental sensitivity^10^, tolerance among individuals^11^, ecological trade-offs and patterns of local adaptation^12,13^. Modelling efforts have demonstrated that some of the main drivers of global change (e.g. temperature and eutrophication) have an antagonistic effect on freshwater community diversity^14^. Yet, empirical assessments of organisms’ response to multiple stressors, exploring how temperature warming may affect organisms’ response to other stressors, is limited (e.g.^15,16^).

Here, we study the impact of high temperature combined with food levels or chemical stressors (insecticides) on the model freshwater species *Daphnia magna*. *D. magna* is a keystone grazer in a wide range of standing freshwater habitats worldwide^17^, and responds to environmental stress via either genetic adaptation or phenotypic plasticity^17,18^. Generally, warming induces plastic responses in life history traits^19,20^, following the temperature-body size rule, according to which organisms are smaller and less fecund in warmer temperatures^21^. Food, both quality and quantity, affects *Daphnia* fitness, reducing growth and fecundity^22^ with food quality playing a stronger role at high food quantity^23,24^. *Daphnia* response to chemicals has been documented for a number of chemicals, including acetylcholinesterase (AChE) and carboxylesterase (CbE) inhibitors which have severe impact on non-target species, affecting their intrinsic growth rate, fecundity and survival^25^. These studies revealed a genetic basis of tolerance showing significant fitness differences among strains and populations^26–28^. Yet, only a handful of studies have investigated the impact of mixtures of stressors on natural populations of *Daphnia* over evolutionary time (e.g.^29,30^).

One way to do this involves examining historical responses to multiple stressors. *Daphnia*’s reproductive strategy offers the unique opportunity to do this via ‘resurrecting’ historical populations from lake sediment by the practise of ‘resurrection ecology’^31^. *Daphnia*’s life cycle alternates sexual recombination with asexual (clonal) reproduction. Sexual recombination results in early stage embryos that arrest their development and remain protected from the environment by a chitin case called an ephippium^32^. Hatching of dormant embryos is achieved under laboratory conditions by exposure to light and temperature stimuli that mimic favourable conditions in the natural environment. Once dormant stages have been revived, *Daphnia* genotypes can be propagated indefinitely in the laboratory via clonal reproduction.

The properties of *Daphnia* provide the opportunity to reveal the relative contribution of plastic and genetic responses to multiple environmental factors through evolutionary time^33,34^. By comparing the responses to environmental change of historical populations to those of their modern descendants, one can investigate how historical exposure to environmental change shapes adaptive responses of modern populations. More generally, by studying populations that originate from the same genetic pool responding to environmental change over time, important insights can be gained into the evolution of fitness traits, which is largely limited to the analysis of spatial populations exposed to differing selection pressures^33–35^.

We studied the impact of warming combined with food levels and carbamate insecticides on *D. magna* populations separated in time, resurrected from a lake with a well-known history of anthropogenic impact^36,37^. This allowed us to understand the evolution of life history traits over five decades and to assess the impact of historical exposure to stress on population responses to recurring and novel stress. On the three resurrected populations, we performed common garden experiments to test five hypotheses:

1. The effect of temperature varies among populations as a result of thermal plasticity^19,20,38^. Higher temperature reduces size and reproductive success of animals^39^.
2. Temperature and non-limiting resources have an antagonistic effect (opposite effect in the individual stressors), which results in a negligible impact of temperature on life history traits^40,41^.
3. Temperature and food limitation have a synergistic (greater than individual stressors) effect on life history traits as a result of higher metabolic demands at high temperatures^41,42^.
4. Carbamate insecticides and temperature have an antagonistic effect as some pesticides display higher volatilization and degradation at higher temperatures^43^. Because of this antagonism, concentrations of pesticides being equal, the impact of carbamate insecticides on fitness is expected to be comparatively higher at lower temperature.
5. Populations that have been exposed to environmental stress prior to dormancy express higher fitness when re-exposed to the same stress^44^. For example, we predict the effect of temperature to be less severe in the most recent population, because of a microevolutionary response to temperature increase^45,46^.

The results of the common garden experiments were validated in competition experiments, in which we assessed population’s relative fitness after exposure to two combinations of stressors and to temperature alone. The competition and the common garden experiments provided insights into the predictability of population response to multiple stressors in light of their historical exposure to environment stress.

## Methods

### Study system

Our study system is Lake Ring, a well characterized shallow mixed lake (without thermocline stratification) located in a typical peri-urban landscape in Jutland, Denmark (55°57′51.83″N, 9°35′46.87″E)^47^. In the late 1950s, sewage inflow from a nearby town dramatically increased nutrient level in the lake resulting in eutrophication^37^. The sewage inflow was diverted at the end of the 1970s, but this period coincided with agricultural land use intensification (>1980), leading to substantial pesticide and herbicide leaching in the lake^37^. The lake was also stocked with white fish between 1989 and 1990 to study the impact of predation on the invertebrate community^36^. Finally, the lake partially recovered from hyper-eutrophication in modern times (>1999s) but, as with every lake in Europe, it experienced an increase in average ambient temperature^48^. A sedimentary archive was collected from Lake Ring in 2004 with a piston corer of 6 cm internal diameter as described in^49^; the core was sliced in layers of 0.5 cm and stored in dark and cold (4 °C) conditions. A radiometric chronology of this sediment was completed in 2015 by ENSIS Ltd (UCL London) following standard protocols^50^, and provided an accurate dating of the sediment to the year 1900. Dating of sediment was conducted by direct gamma assay, using ORTEC HPGe GWL series well-type coaxial low background intrinsic germanium detector. Sediment samples with known radionuclide profiles were used for calibration following^50^.

The sedimentary archive was inspected for dormant *D. magna*. From a total of 923 dormant eggs, 262 dormant *Daphnia* embryos were successfully hatched, following established protocols^49^. Each hatchling is genetically distinct as it is the result of sexual recombination. The hatching success of *D. magna* was, on average, 30.5% across the sedimentary archive, in line with previous studies^51^. Generally, variation in hatching success was not correlated with the age of the sediment (Fig. S1)^49^.

From each of the lake phases–sewage (1960–1970) referred to as the eutrophication phase (EP), pesticide (1975–1985) referred to as the pesticide phase (PP) and recovery (>1999) referred to as the clear water phase (CWP) - we randomly selected ten genotypes (each set of genotypes is referred to hereafter as a population), for a total of 30 genotypes. On clone lines established from the hatchlings, we performed common garden experiments and measured response of fitness-linked life history traits to temperature as a single stressor and in combination with food levels and carbamate insecticide loads. Clonal lineages established from individual hatchlings were maintained in standard laboratory condition (16:8 light: dark regime, 10 °C and 0.4 mg Carbon/L of *Chlorella vulgaris* bi-weekly) for several generations (up to 6 months) to reduce interference from extended dormancy.

The sample size per population of *D. magna* was chosen based on previous results showing that 10 genotypes are representative of the local genetic diversity of *D. magna* populations^52^. The resurrected genotypes are an unbiased representation of the local population genetic diversity as hatching success fluctuated along the sedimentary archive but did not systematically decrease with the age of the sediment^49^. Previous results on the genetic composition of *D. magna* in Lake Ring showed that genetic drift and selection did not have a detectable impact on the neutral genetic diversity over time, measured both on the hatched and unhatched populations of *D. magna* throughout the sedimentary archive^52^. Negligible impact of drift and selection on neutral genetic diversity in the presence of strong environmental selection is ideal to study evolution in life history traits over evolutionary time^52^.

### Paleolimnological and historical profile of Lake Ring

Records of Secchi disk depth (water transparency), total phosphorous and total nitrogen were collected by the County of Vejle in the Jutland peninsula for the years 1971–1999 as part of a monitoring program following standard protocols^53^ (Table S1). Integrated water samples over the entire depth of the lake (5 m) were measured monthly and average values for the summer period (May to October) calculated. A record of pesticides historically sold in Denmark was obtained from the Danish national archives for the period 1955–2010 (Table S2, www.middeldatabasen.dk). According to these records, carbamate insecticides were the third category of most sold insecticides until their ban in early 1990s (Table S2). Temperature records were collected over the past century by the Danish Meteorological Institute at a weather station located 80 km from Lake Ring (Table S1, http://www.dmi.dk/laer-om/generelt/dmi-publikationer/2013/). Because air and water surface temperature have a positive correlation for shallow streams and lakes^54,55^, we used the data from the weather station as estimate of the lake water temperature.

The paleolimnological analysis of the sedimentary archive consisted of quantifying the organic matter content, the *Cladocera* community and the *Daphnia* abundance through time. The organic and carbonate content of the sediment was estimated using loss on ignition, the percentage of sediment weight lost on ignition (LOI)^56^. The procedure for LOI consists of strongly heating (“igniting”) a sediment sample at a specified temperature (550 °C), burning off the organic fraction^57^. LOI is used in limnology as an indirect measure of eutrophication as Carbon is the main component of primary producers and a good estimate of their abundance^58^. The *Cladocera* community composition in each sediment layer was quantified from sub-fossil remains following^59^. In brief, each sample (sediment layer) was heated in a deflocculating agent (KOH) and sieved at 150 and 50 µm. The remains from the two sieves were washed separately and stained with safranin. Sub-samples from each layer were analysed using a compound microscope at x40–400. The chitinous *Cladocera* remains were identified following^60,61^. Changes in *D. magna* abundance were quantified from the count of the dormant *D. magna* embryos recovered from each sediment layer and multiplied for the total lake surface (22.5 hectares).

### Life history traits response to single and multiple stressors

**Figure 1:**
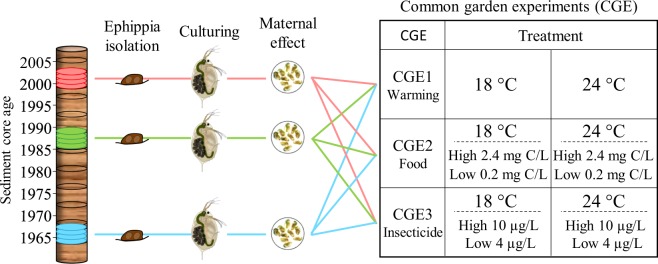
Experimental design. *D. magna* dormant embryos were sampled from a sedimentary archive of Lake Ring. Ten hatchlings from each lake phase (N = 30) were used in three common garden experiments (CGEs) and life history traits (mortality, size at maturity, age at maturity and fecundity) measured after controlling for maternal effect. CGE1: temperature; CGE2: temperature and food levels; CGE3: temperature and insecticide Carbaryl concentrations. Temperatures used are: 18 °C and 24 °C; food levels are 0.2 mg Carbon/L and 2.4 mg Carbon/L; Carbaryl concentrations are 4 μg/L and 10 μg/L.

The impact of temperature in isolation and in combination with food levels and carbamate insecticides, was assessed via life history traits across the 30 genotypes resurrected from Lake Ring, 10 genotypes per population separated in time, in three common garden experiments (Fig. 1).

In common garden experiment 1 (CGE1) we measured life history response to high temperature (24 ± 1 °C) as compared to a control temperature (18 ± 1 °C); 24 °C represents an increase of 6 °C of the current lake temperature; food levels in this experiment were non-limiting (0.8 mg Carbon/L of *Chlorella vulgaris*). In common garden experiment 2 (CGE2), the two temperatures were crossed with two nutrient levels: 0.2 mg Carbon/L and 2.4 mg Carbon/L. For this experiment, we use food quantity to mimic changes in total primary producer’s biomass, because it is a more realistic ecological approximation of eutrophication as a complex environmental stress, which includes change in turbidity and oxygen in addition to food quality. In common garden experiment 3 (CGE3), the two temperatures were crossed with two concentrations of a carbamate insecticide (commercial name Carbaryl, Pestanatal): 4 µg/L and 10 µg/L; in this experiment, non-limiting food levels were used (0.8 mg Carbon/L of *C. vulgaris*). We decided to use Carbaryl, as it was previously shown to have a measurable impact on *Daphnia* fitness^26–28,62,63^ and it was one of the most common brands of carbamate insecticides sold in the 1980s (Table S2). Concentrations of food and Carbaryl imposing sub-lethal effects on *D. magna* life history traits were identified in the study populations using pilot experiments (Appendix 1).

Prior to starting the CGEs, the genotypes were acclimated and synchronized for two generations in common garden conditions (16:8 light: dark regime, 16 ± 1 °C and fed 0.8 mg Carbon/L of *C. vulgaris* daily) to reduce interference from maternal effects. After two generations in these conditions, 24–48 hour old juveniles from the second or following broods were randomly assigned to the experimental exposures in which fitness-linked life history traits were measured in the time spanning an individual life cycle (until release of the second brood). For each clone of the same genotype across the three CGEs we measured size at maturity (distance between the head and the base of the tail spine), age at maturity (first time eggs were observed in the brood chamber), fecundity (total number of offspring released summing first and second brood), and mortality. For size at maturity, all animals were measured after releasing their first brood into the brood pouch using image J software (https://imagej.nih.gov/ij/index.html). We used life history trait measurements to make inferences at population level throughout the study, using genotypes as replicates per population.

Mortality rates per population were calculated with a survival model fit via the psm function in the rms R package V.3.3^64^. A separate model was fitted to each treatment, in which the day of mortality and mortality event were combined as the dependent variables (e.g. censoring); population and temperature were treated as fixed effects. All mortality curves were plotted using the survplot function form rms package in R v.3.3.3^64^.

We quantified the effects of population, treatment and their interaction on individual life history traits measured in the three CGEs using linear mixed models (LMMs) in R v.3.3.3^64^. Genotype was fit as a random effect nested within population. Because the populations separated in time originate from the same genetic pool and genetic drift is negligible^52^, a significant population term indicates genetic differences among populations. Differences in mean trait values between temperature, nutrient or carbamate treatments, after controlling for maternal effects, are the expression of plasticity, an environmental effect. If the effect of the treatments (environments) differed significantly among populations (genetic effect), we would have evidence for a P (population) × E (treatment) interaction. We visualized the main effects and the population x treatment interactions via population reaction norms, which describe the pattern of phenotypic expression across treatments^65^. All evidence of interactions or main effects were assessed via Type II analysis of deviance tables via the Anova function in the car library for R v.3.3.3^66^.

### Competition experiments

To test the relative fitness of the three populations of *D. magna* in the presence of multiple stressors, we performed competition experiments in 10 L mesocosms. In these experiments, we quantified fitness differences among populations by measuring changes in each population frequency after four weeks of exposure to multiple stressors as compared to an initial inoculum, in which population frequencies were identical. We measured population response to high temperature (24 °C) combined with resource limitation (low food, 0.2 mg Carbon/L) and high temperature combined with high concentrations of Carbaryl (10 µg/L). Previously, Cambronero and Orsini (2018) performed a competition experiment using the same genotypes to quantify the impact of high temperature as a single stressor^49^. Here, we used data from this previous experiment to summarize population response to a single stress and multiple stressors.

Prior to the competition experiments all genotypes were cultured for two generations in the following conditions to control for maternal effects and synchronize reproduction: 20 °C; long photoperiod (16:8 light:dark regime); feed daily with 0.8 mg Carbon/L of *C. vulgaris;* medium was renewed every second day. Five 24–48 h old juveniles from the second brood of the third generation from 7 genotypes of each population were randomly assigned to 10 L mesocosms for a total of 105 animals per mesocosm [5 juveniles × 7 genotypes × 3 populations]. Each experiment was run in triplicate. The mesocosms were exposed to the experimental conditions for four weeks (≥3 clonal generations). To simulate population dynamics that *Daphnia* may encounter in the natural environment (e.g. mortality by competition and predation), we culled 10% (1 L) of the volume of each mesocosm at regular intervals on day 10, 17 and 24, after thorough mixing, removing medium and a random number of individuals collected in the culled medium following^20,33^. The volume of culled medium was replaced with fresh medium.

At the end of the fourth week, 32 (10%) animals from each mesocosm (N = 192) were randomly sampled to assess changes in population frequency as compared to the initial inoculum. To measure changes in population frequency, we used a panel of 13 polymorphic microsatellites arranged in two multiplexes (M01 and M05). These loci are part of a panel of 84 microsatellites previously developed for *D. magna*^67,68^. Genomic DNA was extracted from single individuals using AGENCOURT® DNAdvance (Beckman Coulter) kit with minor modifications. The samples were amplified using established protocols^67,68^ and genotyped on an ABI3032. Fragment analysis was conducted with Genemapper (Thermo Fisher Scientific) using LIZ500 (Thermo Fisher Scientific) as size standard. At the end of the experiment, we quantified changes in the frequency of each population between the inoculum and the end of the experiment with a chi-squared test using the “stats” package in R v.3.3.3^69^. A non-significant change in population frequency after four weeks of exposure to either single or multiple stressors indicates that the population was not affected by the stressors and maintained a frequency similar to the initial inoculum. Conversely, a significant decrease or increase in frequency as compared to the initial inoculum indicates a negative or positive effect on population fitness, respectively. For the analysis of population frequency in response to high temperature we used previously generated data^33^.

## Results

### Life history traits response to single and multiple stressors

We investigated the impact of temperature alone (CGE1), temperature and food levels (CGE2) and temperature and insecticide Carbaryl loads (CGE3) on fitness-linked life history traits.

#### CGE1 (Temperature)

**Figure 2:**
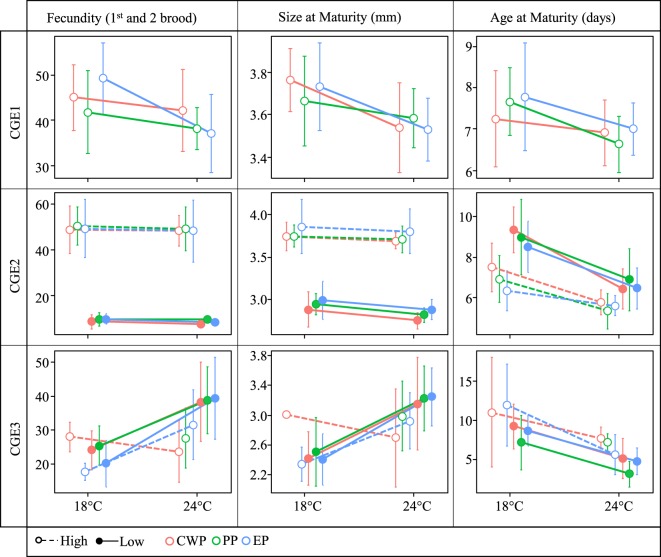
Univariate reaction norms. Univariate responses to temperature (CGE1), temperature combined with food levels (CGE2) and temperature combined with loads of the insecticide Carbaryl (CGE3) in the three populations of *D. magna* resurrected from Lake Ring. Population reaction norms based on population means (n = 10) and SD, are shown for three life history traits. Responses in the life history traits to temperature, temperature combined with food levels and temperature combined with Carbaryl loads are shown. Only mortality data are available for the PP population at high concentrations of Carbaryl as the population experienced 100% mortality in this treatment three days after exposure (Fig. S1). The populations are colour coded as in Fig. 2.

The effect of temperature on all traits did not vary by population (Table 1 - CGE1, P × T). Furthermore, we detected no difference among populations in trait means (Table 1 - CGE1, P). Increasing temperature caused a decrease in mean size at maturity, age at maturity and fecundity (Fig. 2 - CGE1) but did not affect mortality (Fig. S2).

**Table 1:** Analysis of variance.

CGE1	Fecundity		Size at maturity		Age at maturity		Mortality	
	Chisq	P-val	Chisq	P-val	Chisq	P-val	Chisq	P-val
Population (P)	1.422	0.491	0.074	0.964	1.753	0.416	3.765	0.152
Temperature (T)	8.563	**<0.001**	13.061	**<0.001**	5.102	**0.024**	2.043	0.153
P × T	3.494	0.174	1.908	0.385	1.162	0.559	4.121	0.127
**CGE2**								
Population (P)	0.470	0.791	4.816	0.090	2.717	0.257	3.865	**0.014**
Temperature (T)	0.552	0.457	12.543	**<0.001**	100.929	**<0.001**	0.005	0.946
Food(F)	1033.209	**<0.001**	1202.270	**<0.001**	62.856	**<0.001**	0.415	0.519
P × T	0.034	0.983	0.178	0.915	4.343	0.114	8.472	**0.014**
P × F	0.102	0.951	1.322	0.516	1.419	0.492	0.283	0.868
T × F	0.001	0.978	1.707	0.191	6.604	**0.010**	3.494	0.062
P × T × F	0.153	0.926	0.140	0.932	0.768	0.681	3.365	0.186
**CGE3**								
Population (P)	0.367	0.832	0.560	0.756	6.054	**0.048**	2.218	0.330
Temperature (T)	48.440	**<0.001**	67.951	**<0.001**	52.086	**<0.001**	11.494	**<0.001**
Insecticide (I)	13.619	**<0.001**	2.651	0.104	15.185	**<0.001**	82.991	**<0.001**
P × T	3.457	0.178	1.996	0.369	0.158	0.924	0.462	0.794
P × I	0.283	0.868	1.255	0.534	1.747	0.417	1.662	0.436
T × I	4.045	**0.044**	5.189	**0.023**	0.249	0.618	4.857	**0.028**
P × T × I	1.365	0.243	1.656	0.198	1.039	0.308	0.463	0.793

ANOVAs per single life history trait (mortality, fecundity, size and age at maturity) calculated for the three common garden experiments testing for the effect of population, treatment and their interaction terms. The populations are as in Fig. 1, each comprising 10 *Daphnia magna* genotypes and representing different time periods. CGE1 - Temperature treatment; CGE2 - Temperature and food levels; CGE3 - temperature and insecticide Carbaryl. Significant *P-values* are shown in bold.

#### CGE2 (Temperature and food)

The three way interaction (food x temperature x population) was not significant in the temperature and food experiment (Table 1 - CGE2, P × F × T). The effect of temperature did not vary by population, except for mortality (Table 1 - CGE2, P × T). Similarly, the effect of food did not vary by population (Table 1 - CGE2, P × F). We detected no difference among populations in trait means, except for mortality (Table 1 - CGE2, P). Increasing temperature caused a decrease in size and age at maturity (Fig. 2 - CGE2). Food limitation caused lower fecundity, smaller size at maturity and a delay in maturation (Fig. 2 - CGE2). The effect of food on trait means did not vary by temperature (Table 1 - CGE2, T × F).

#### CGE3 (Warming and insecticide Carbaryl)

There was no evidence that an interaction between insecticide and population varied by temperature in the temperature and Carbaryl experiment (Table 1 – CGE3, P x F x T). The effect of temperature did not vary by population in any of the life history traits (Table 1 - CGE3). Similarly, the effect of insecticide did not vary by population (Table 1 - CGE3, P × I). We detected no difference among populations in trait means, except for age at maturity (Table 1 - CGE3, P). The effect of insecticide on trait means varied significantly by temperature in all traits, except for age at maturity (Table 1 - CGE3, T × I). Increasing temperature in low Carbaryl caused an increase in fecundity, a larger size at maturity, and an earlier age at maturation (Fig. 2 - CGE3). Increasing temperature in high Carbaryl had a population-dependent effect; the CWP population experienced a decrease in fecundity and a smaller size at maturity, whereas the EP population experienced an increase in fecundity and a larger size at maturity (Fig. 2 - CGE3). Both EP and CWP experienced earlier maturation (Fig. 2 - CGE3). The PP population experienced 100% mortality in high Carbaryl (Fig. S2).

### Competition experiments

**Figure 3:**
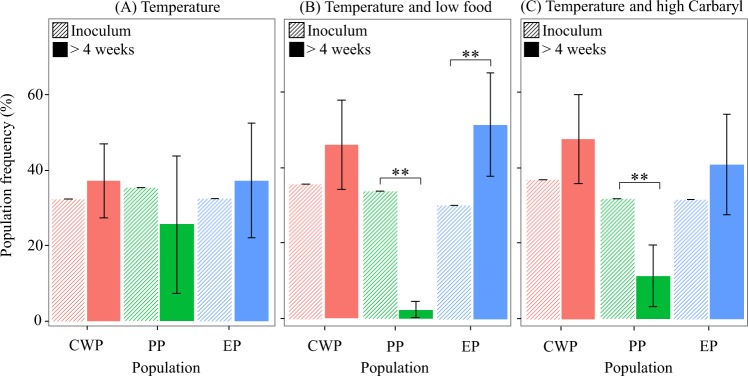
Competition experiments. Population frequencies with variance are shown after four weeks of exposure (‘>4 weeks’) to (**A**) temperature, (**B**) temperature and limiting food levels, and (**C**) temperature and high Carbaryl as compared to an equal starting inoculum of the three populations (‘inoculum’). The starting inoculum had no variance as all mesocosms were inoculated with equal number of genotypes. Population codes are as in Fig. 2. Data for the temperature experiment are from^33^.

In our previous work^49^, we found that high temperature did not alter the frequency of the genotypes from any of the three populations [χ^2^_CWP_ = 0.33, p = 0.57; χ^2^_PP_ = 1.54, p = 0.21; χ^2^_EP = _0.34, p = 0.56] (Fig. 3A).

Exposure for four weeks to high temperature combined with resource limitation (low food) did not result in a significant change of genotype frequency from the CWP population [χ^2^_CWP_ = 1.29, p = 0.26], whereas it increased the frequency of genotypes from the EP population [χ^2^_EP_ = 5.68, p = 0.02] and it decreased the frequency of genotypes from the PP population [χ^2^_EP_ = 27.19, p = 0.001] (Fig. 3B).

Exposure for four weeks to high temperature and high Carbaryl had no effect on the frequency of genotypes from the CWP and the EP populations [χ^2^_CWP_ = 1.40, p = 0.24; χ^2^_EP_ = 1.22, p = 0.27], whereas it decreased the frequency of those from the PP population [χ^2^_PP_ = 9.50, p = 0.02] (Fig. 3C).

### Environmental profile of Lake Ring

**Figure 4:**
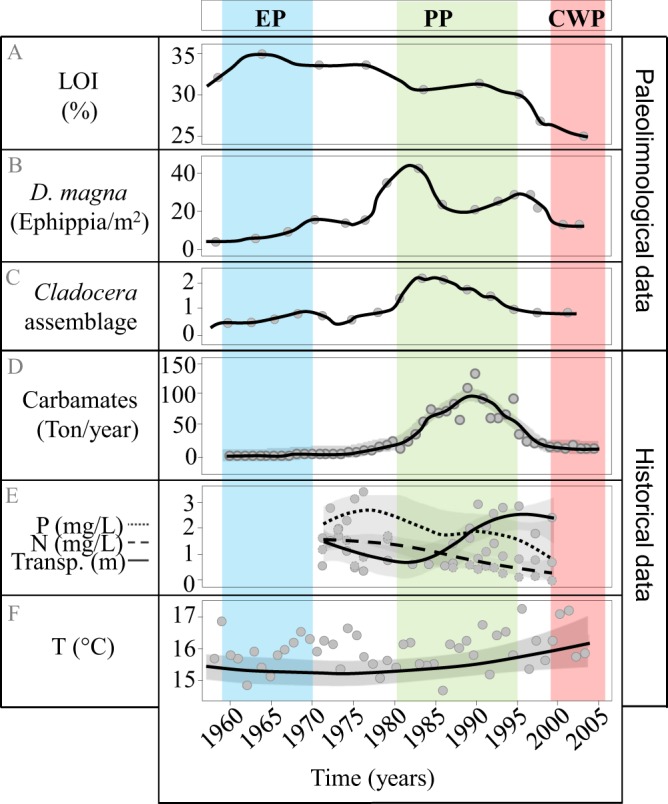
Lake Ring historical profile. Paleolimnological and historical data are shown for Lake Ring. (**A**) loss on ignition (LOI, %); (**B**) *Daphnia magna* ephippia abundance (m^2^); (**C**) *Cladocer*a assemblage based on 33 taxa after excluding *Daphnia magna*; (**D**) Carbaryl usage (tons per year); (**E**) average summer air temperatures (June-August); (**F**) total phosphorus (P, mg/L), total nitrogen (N, mg/L), and transparency calculated as Secchi disk depth (m) for the period 1970–1999. EP - eutrophic population (blue), PP - pesticide population (green), and CWP - clear-water population (red). Grey areas represent 95% confidence intervals.

Historical records documenting sewage inflow in Lake Ring matched a high level of primary production in the paleolimnological analysis - high LOI (Fig. 4A, EP). *D. magna* abundance was low at the beginning of the 1960s and increased towards the 1970s (Fig. 4B). The *Cladocera* community followed a similar trend (Fig. 4C). From the 1970s onwards, primary production fluctuated but remained overall high until the mid-1990s (Fig. 4A, LOI). In the early 1970s, high LOI was matched by low transparency, high total phosphorus and total nitrate (Fig. 4E). The *Daphnia* population and the *Cladocera* community reached a peak of abundance in the early 1980s (Fig. 4B,C). In 1985, the use of carbamate insecticides was highest (Fig. 4D). Coincident with the peak of carbamates, the *D. magna* population declined (Fig. 4B), even though primary production was high (Fig. 4A). In the 1990s, white fish were stocked in the lake for a year, imposing a short-lived negative effect on *D. magna* population (Fig. 4B,C), which subsequently recovered. With the diversion of sewage and decrease in agricultural land use^70^ in modern times, a decline in primary production was observed after the 1990s coinciding with a lower presence of *Daphnia* population and the *Cladocera* community. Consistent with lower primary production (Fig. 2B,C), a decrease in total phosphate and total nitrate and an increase in water transparency were observed (Fig. 4E). Finally, a modest but steady increase (∼1 °C) in environmental temperature was recorded over the five decades studied (Fig. 4F). According to these records the oldest population was sampled from the coldest period and the most recent population from the warmest period of the 21^st^ century^48^.

## Discussion

Our first objective was to assess four hypotheses on the impact of temperature, food levels and carbamate insecticides, in isolation and in combination, on *Daphnia magna* life history traits. Our second objective was to understand how historical exposure to environmental stress impacts population level response to recurring and novel stress.

Our first hypothesis was that the populations analysed expressed thermal plasticity in response to warming. As a corollary of this hypothesis, we expected that higher temperature induced smaller size at maturation and lower fecundity. We observed thermal plasticity in the three populations exposed to temperature alone (CGE1), and non significant genetic differences among populations. This finding was confirmed by the competition experiment, in which the three populations showed comparable competitive abilities in response to warming. At higher temperature, genotypes reached maturation faster at a smaller size and were less fecund, following the temperature-body size rule, according to which organisms are smaller and reproduce less in warmer temperatures^21^. These results suggest trade-offs among life history traits, where a decline in fecundity is compensated by faster maturation and smaller body size^71^. Reduction in body size of grazers may have far reaching consequences in aquatic food webs by altering the predator-prey^72^ and the grazer-primary producer’s^73^ dynamics (see^14^ for a review on the impact of temperature on the complexity of food webs). Changes in grazers’ body size have a direct impact on visual predators (e.g. fish). Moreover, because high temperature induces higher primary production but lower fecundity in the grazers resulting in lower population growth rate, warming influences biomass of grazers and their primary producers in opposite directions. Overall, our results on *Daphnia* response to temperature indicate a potential negative impact of global warming on freshwater communities, confirming modelling predictions^14,74^.

Our second hypothesis was that warming and high food levels (non-limiting resources) had antagonistic effects, as higher resource availability compensated for higher metabolic demands associated with high temperature. In the common garden experiment testing the impact of temperature and high food level (CGE2), we observed no decrease in fecundity and size at maturity with temperature; this trend was reversed in the temperature only exposure (CGE1). These results suggest that in a global change scenario, in which ecosystem productivity is high, the potentially negative effect of high temperature on *Daphnia*, linked to higher metabolic demands, can be partially mitigated by high resource availability. However, high productivity is generally associated with eutrophication, leading to a shift from algae to cyanobacterial blooms, with a negative cascading effect on higher trophic levels^75^.

Our third hypothesis was that temperature and food limitation had a synergistic or additive effect with warming. We observed a synergistic effect of food limitation and warming only on age at maturity (T × F term in the ANOVA analysis). Moreover, populations did not show significant genetic differences in their response to the combined effect of temperature and food limitation. Interestingly, temperature and population interacted significantly in limited food levels, resulting in significantly different population fitness in the competition experiment, in which the frequency of the PP population significantly decreased, and the frequency of the EP population significantly increased over the time of the experiment. This suggests that in presence of a second stressor (e.g. food limitation) temperature may negatively affect population fitness, depending on its genetic background or, possibly, its history of exposure to stress. If these trends were to be confirmed in the wild, the impact of temperature on natural populations may be severely augmented by the co-occurrence of a second stressor, altering the genetic composition of natural populations. By studying the impact of global change using temperature as a *proxy*, we may severely underestimated the effect of warming on the genetic composition of populations and, therefore, on the dynamics of food webs.

Our fourth hypotheses was that temperature and carbamate insecticides had an antagonistic effect because of higher volatilization and degradation of the insecticides at higher temperature^43^. Previous studies on *Daphnia* showed a negative effect of carbamate insecticides on individual performance^28^. However, both synergistic and antagonistic effects of a second stressor were observed on carbamate-exposed *Daphnia*; exposure to carbamates increased the impact of parasite infection^28^ but not of predation^63^. We observed a more severe effect of the carbamate insecticide at 18 °C compared to 24 °C on all life history traits, and in particular, on mortality. We observed a net increase in fecundity and larger size at maturity in animals exposed to carbamates at 24 °C as compared to the same animals at 18 °C. The net fitness advantage of higher volatilization of carbamate insecticide was obvious in respect to the temperature only experiment, in which fecundity was lower and size smaller at 24 °C.

Hence, the common garden experiment (CGE3) validated the hypothesis of antagonistic effect of temperature and insecticide for low concentration of the insecticide. These results suggest that the adverse effect of some insecticides on non-target species may be mitigated under warming. However, the mitigating effect of temperature on the insecticide was only observed for low concentrations. In fact, the mitigating effect of temperature on high concentration of carbamates was evident only on the EP population, whereas warming and insecticide had a synergistic effect on the CWP population, in which we observed a decrease in fecundity and a smaller size at maturity at 24 °C compared to 18 °C. The competition experiment confirmed significant differences in population competitive abilities in the presence of a high concentration of carbamate and warming, suggesting a population-dependent response to multiple stressors that may be driven by historical exposure to stress. We discuss the possible influence of historical exposure on population fitness below.

The paleolimnological analysis of the sedimentary archive suggested that the *D. magna* population responded negatively to pesticides. Conversely, *Daphnia* and the *Cladocera* community abundance did not change appreciably during the period of sewage inflow (1960–1970), suggesting that this event did not have an impact on species abundance. However, it is noteworthy that both the *Daphnia* population and the *Cladocera* community are less abundant during the eutrophication phase than later phases. Although a causative link among these environmental factors and the *Daphnia* population dynamics cannot be established at this point, the paleolimnological analysis confirmed that *D. magna* from Lake Ring were indeed exposed to a number of environmental factors, including pesticides, temperature and nutrient levels and that they persisted over time, possibly via adaptive or microevolutiaonary responses. In light of this evidence we hypothesized less impact of temperature on the most recent population, because of a microevolutionary response to temperature increase. This hypothesis is supported by previous studies showing evolution of the critical thermal maximum (CT_max_) in populations of *D. magna*, including the population studied here, over multiple decades^45,46,76^. This hypothesis was not validated in the present study, as we did not observe genetic differences among populations in response to temperature or higher competitive ability of the most recent population in the temperature competition experiment. Microevolutionary responses in physiological traits, such as CT_max_, and lack thereof in life history traits may be explained by the traits being under different evolutionary constraints.

In the context of historical exposure positively affecting population response to recurring stress, we expected the impact of food limitation to be less severe in the clear-water population and for the eutrophic population to have higher performance in high food levels. Generally, the three populations responded with a similar degree of plasticity across life history traits to resource limitation and high food level combined with temperature in the common garden experiments. Moreover, in the competition experiment, the clear water and the eutrophic populations showed comparable competitive abilities when exposed to resource limitation and warming, irrespective of their historical exposure to food levels. Unpredicted patterns were also observed in the carbamate and warming exposure, in which the pesticide population, which experienced pesticide exposure prior to dormancy, was, in fact, the one with the lowest fitness. These observations may be the result of hatching bias in the resting egg bank and/or gene flow from other populations. However, we can rule out both biases. A recent study of the *Daphnia* populations resurrected from three biological archives, sampled from three lakes and including Lake Ring, showed that dormant egg banks can be interrogated to obtain an unbiased measure of genetic diversity over time, demonstrating that temporal neutral genetic stability was directly comparable between hatched and unhatched populations^52^. The same study showed no detectable changes over time of the allelic composition of the populations, suggesting that the establishment success of migrants is generally negligible in *Daphnia* populations^52^.

Negligible impact of migrant’s establishment in *D. magna* has been previously explained with the monopolization hypothesis, according to which local genetic adaptation of initial colonizing genotypes results in a reduction of gene flow that fosters the persistence of founder effects^68,77,78^. The lower fitness of the pesticide population in the common garden and competition experiments as compared to the other two populations may be explained by the negative effect of historical pesticide exposure that is magnified in the presence of temperature or food limitation, supporting the hypothesis of synergism between chemical pollution and other stressors. Synergism between pesticides and other stressors is well documented^28,41,79,80^.

Overall, our study showed that the impact of multiple stressors can be empirically estimated, suggesting that an overarching predictive approach on how combinations of stressors affect natural ecosystems may be achievable. These data have shown that the impact of temperature on natural populations could be either more severe or mitigated in the presence of other environmental factors. For example, the effect of temperature on population fitness was mitigated in the presence of high food levels, but was more severe in the presence of a high carbamate insecticide. Given these interactions, the use of temperature as single *proxy* for species response to global change may lead to over- and under-estimates of species evolvability and persistence.

## Electronic supplementary material

Supplementary Information

## Data Availability

Data associated with this study are deposited in the DRYAD databank at the following entry: DOI: doi:10.5061/dryad.cp45ht6 and https://doi.org/10.5061/dryad.5k6t6.

## References

[1] Jenkins M (2003). Prospects for biodiversity. Science.

[2] Hallmann Caspar A., Sorg Martin, Jongejans Eelke, Siepel Henk, Hofland Nick, Schwan Heinz, Stenmans Werner, Müller Andreas, Sumser Hubert, Hörren Thomas, Goulson Dave, de Kroon Hans (2017). More than 75 percent decline over 27 years in total flying insect biomass in protected areas. PLOS ONE.

[3] Ormerod SJ, Dobson M, Hildrew AG, Townsend CR (2010). Multiple stressors in freshwater ecosystems. Freshwater Biol.

[4] WWF. Living Plant Report 2014: Species and Spaces, People and Places (Gland, Switzerland, 2014).

[5] Ansari, A. A., Gill, S. S., Lanza, G. R. & Rast, W. *Eutrophication: Causes, Consequences and Control* (Springer, 2011).

[6] Foley JA (2005). Global consequences of land use. Science.

[7] Sala OE (2000). Global biodiversity scenarios for the year 2100. Science.

[8] Spaak JW (2017). Shifts of community composition and population density substantially affect ecosystem function despite invariant richness. Ecol Lett.

[9] Wu, P. P. Y. *et al*. Timing anthropogenic stressors to mitigate their impact on marine ecosystem resilience. *Nature Communications* **8**, 10.1038/s41467-017-01306-9 (2017). 10.1038/s41467-017-01306-9 PMC5665875 29093493

[10] Venâncio Cátia, Ribeiro Rui, Soares Amadeu, Lopes Isabel (2016). Multiple Stressor Differential Tolerances: Possible Implications at the Population Level. PLOS ONE.

[11] Baert JM, Janssen CR, Sabbe K, De Laender F (2016). Per capita interactions and stress tolerance drive stress-induced changes in biodiversity effects on ecosystem functions. Nat Commun.

[12] Kneitel JM, Chase JM (2004). Trade-offs in community ecology: linking spatial scales and species coexistence. Ecology Letters.

[13] Bennett AF, Lenski RE (2007). An experimental test of evolutionary trade-offs during temperature adaptation. P Natl Acad Sci USA.

[14] Binzer A, Guill C, Rall BC, Brose U (2016). Interactive effects of warming, eutrophication and size structure: impacts on biodiversity and food-web structure. Glob Chang Biol.

[15] Brans KI (2017). The heat is on: Genetic adaptation to urbanization mediated by thermal tolerance and body size. Global Change Biol.

[16] Christensen MR (2006). Multiple anthropogenic stressors cause ecological surprises in boreal lakes. Global Change Biol.

[17] Miner BE, De Meester L, Pfrender ME, Lampert W, Hairston NG (2012). Linking genes to communities and ecosystems: Daphnia as an ecogenomic model. P Roy Soc B-Biol Sci.

[18] De Meester L, Van Doorslaer W, Geerts A, Orsini L, Stoks R (2011). Thermal Genetic Adaptation in the Water Flea Daphnia and its Impact: An Evolving Metacommunity Approach. Integr Comp Biol.

[19] Mitchell SE, Lampert W (2000). Temperature adaptation in a geographically widespread zooplankter, Daphnia magna. J Evolution Biol.

[20] Van Doorslaer W, Stoks R, Duvivier C, Bednarska A, De Meester L (2009). Population dynamics determine genetic adaptation to temperature in Daphnia. Evolution.

[21] Atkinson D, Sibly RM (1997). Why are organisms usually bigger in colder environments? Making sense of a life history puzzle. Trends Ecol Evol.

[22] Pietrzak B, Grzesiuk M, Bednarska A (2010). Food quantity shapes life history and survival strategies in Daphnia magna (Cladocera). Hydrobiologia.

[23] Sarpe D, Domis LND, Declerck SAJ, van Donk E, Ibelings BW (2014). Food quality dominates the impact of food quantity on Daphnia life history: possible implications for re-oligotrophication. Inland Waters.

[24] Lukas M, Wacker A (2014). Daphnia’s dilemma: adjustment of carbon budgets in the face of food and cholesterol limitation. J Exp Biol.

[25] Altshuler I (2011). An Integrated Multi-Disciplinary Approach for Studying Multiple Stressors in Freshwater Ecosystems: Daphnia as a Model Organism. Integr Comp Biol.

[26] Jansen M (2015). Experimental evolution reveals high insecticide tolerance in Daphnia inhabiting farmland ponds. Evol Appl.

[27] Jansen M, De Meester L, Cielen A, Buser CC, Stoks R (2011). The interplay of past and current stress exposure on the water flea Daphnia. Funct Ecol.

[28] Jansen M, Stoks R, Coors A, Van Doorslaer W, De Meester L (2011). Collateral damage: rapid exposure-induced evolution of pesticide resistance leads to increased susceptibility to parasites. Evolution.

[29] Altshuler I, McLeod AM, Colbourne JK, Yan ND, Cristescu ME (2015). Synergistic interactions of biotic and abiotic environmental stressors on gene expression. Genome.

[30] Heugens EH (2006). Population growth of Daphnia magna under multiple stress conditions: joint effects of temperature, food, and cadmium. Environ Toxicol Chem.

[31] Kerfoot WC, Weider LJ (2004). Experimental paleoecology (resurrection ecology): Chasing Van Valen’s Red Queen hypothesis. Limnol Oceanogr.

[32] Ebert, D. *Ecology, epidemiology, and evolution of parasitism in Daphnia*. (National Library of Medicine (US), National Center for Biotechnology, 2005).

[33] Cambronero, C. M., Zeis, B. & Orsini, L. Haemoglobin-mediated response to hyper-thermal stress in the keystone species Daphnia magna. *Evol Appl* in press (2017). 10.1111/eva.12561 PMC5748520 29302276

[34] Stoks Robby, Govaert Lynn, Pauwels Kevin, Jansen Bastiaan, De Meester Luc (2015). Resurrecting complexity: the interplay of plasticity and rapid evolution in the multiple trait response to strong changes in predation pressure in the water fleaDaphnia magna. Ecology Letters.

[35] Govaert L, Pantel JH, De Meester L (2016). Eco-evolutionary partitioning metrics: assessing the importance of ecological and evolutionary contributions to population and community change. Ecol Lett.

[36] Berg S, Jeppesen E, Sondergaard M, Mortensen E (1994). Environmental-effects of introducing Whitefish, Coregonus- Lavaretus (L), in Lake Ring. Hydrobiologia.

[37] Michels, H. *Micro-evolutionary response of Daphnia magna to changes in biotic stress associated with habitat degradation and restoration of a shallow lake* Biology thesis, University of Leuven, (2007).

[38] Van Doorslaer W, Stoks R, Jeppesen E, De Meester L (2007). Adaptive microevolutionary responses to simulated global warming in Simocephalus vetulus: a mesocosm study. Global Change Biol.

[39] Atkinson D (1994). Temperature and organism size: A biological law for ectotherms?. Advances in Ecological Research.

[40] Doyle SA, Saros JE, Williamson CE (2005). Interactive effects of temperature and nutrient limitation on the response of alpine phytoplankton growth to ultraviolet radiation. Limnol Oceanogr.

[41] Jackson MC, Loewen CJG, Vinebrooke RD, Chimimba CT (2016). Net effects of multiple stressors in freshwater ecosystems: a meta-analysis. Global Change Biol.

[42] Giebelhausen B, Lampert W (2001). Temperature reaction norms of Daphnia magna: the effect of food concentration. Freshwater Biol.

[43] Lima MPR, Cardoso DN, Soares AMVM, Loureiro S (2015). Carbaryl toxicity prediction to soil organisms under high and low temperature regimes. Ecotoxicology and Environmental Safety.

[44] Desai MM (2009). Reverse evolution and evolutionary memory. Nat Genet.

[45] Geerts A. N., Vanoverbeke J., Vanschoenwinkel B., Van Doorslaer W., Feuchtmayr H., Atkinson D., Moss B., Davidson T. A., Sayer C. D., De Meester L. (2015). Rapid evolution of thermal tolerance in the water flea Daphnia. Nature Climate Change.

[46] Jansen M (2017). Thermal tolerance in the keystone species Daphnia magna-a candidate gene and an outlier analysis approach. Mol Ecol.

[47] Sayer C, Davidson A, Jones JI (2010). Seasonal dynamics of macrophytes and phytoplankton in shallow lakes: a eutrophication-driven pathway from plants to plankton?. Freshwater Biol.

[48] IPCC. Summary for policymakers 1–32 (Cambridge, United Kingdom and New York, NY, USA, 2014).

[49] Cambronero, C., M. & Orsini, L. Resurrection of dormant Daphnia magna: protocol and applications. *JoVE* in press (2018). 10.3791/56637 PMC5908671 29443016

[50] Appleby, P. G. *Chronostratigraphic techniques in recent sediments*. Vol. 1 (Kluwer Academic Publisher, 2001).

[51] Schwartz SS, Hebert PDN (1987). Methods for the Activation of the Resting Eggs of Daphnia. Freshwater Biol.

[52] Orsini L (2016). Temporal genetic stability in natural populations of the waterflea Daphnia magna in response to strong selection pressure. Molecular Ecology.

[53] Søndergaard M (1990). Phytoplankton biomass reduction after planktivorous fish reduction in a shallow, eutrophic lake: a combined effect of reduced internal P-loading and increased zooplankton grazing. Hydrobiologia.

[54] Preudhomme, E. B. & Stefan, H. G. Relationship between water temperatures and air temperatures for central U.S. streams. (University of Minnesota, St. Anthony Falls hydraulicLaboratory, Duluth, Minnesota, 1992).

[55] Livingstone DM, Lotter AF (1998). The relationship between air and water temperatures in lakes of the Swiss Plateau: a case study with palæolimnological implications. Journal of Paleolimnology.

[56] Heiri O, Lotter AF, Lemcke G (2001). Loss on ignition as a method for estimating organic and carbonate content in sediments: reproducibility and comparability of results. Journal of Paleolimnology.

[57] Santisteban JI (2004). Loss on ignition: a qualitative or quantitative method for organic matter and carbonate mineral content in sediments?. Journal of Paleolimnology.

[58] Downing J. A., Cole J. J., Middelburg J. J., Striegl R. G., Duarte C. M., Kortelainen P., Prairie Y. T., Laube K. A. (2008). Sediment organic carbon burial in agriculturally eutrophic impoundments over the last century. Global Biogeochemical Cycles.

[59] Davidson T, Sayer C, Perrow M, Bramm M, Jeppesen E (2007). Are the controls of species composition similar for contemporary and sub-fossil cladoceran assemblages? A study of 39 shallow lakes of contrasting trophic status. Journal of Paleolimnology.

[60] Flossner, D. *Die Haplopoda und Cladocera (ohne Bosminidae) Mitteleuropas* (Backhuys Publishers, 2000).

[61] Fryer G (1985). Crustacean diversity in relation to the size of water bodies: some facts and problems. Freshwater Biol.

[62] Jansen M, Coors A, Stoks R, De Meester L (2011). Evolutionary ecotoxicology of pesticide resistance: a case study in Daphnia. Ecotoxicology.

[63] Jansen M, Stoks R, Coors A, De Meester L (2010). No evidence for a cost of selection by carbaryl exposure in terms of vulnerability to fish predation in Daphnia magna. Hydrobiologia.

[64] R: A language and environment for statistical computing (Vienna, Austria, 2017).

[65] Roff, D. A. *Phenotypic Plasticity and Reaction Norms* (Springer, 1997).

[66] Fox, J. & Weisberg, S. *An R Companion to Applied Regression, Second Edition* (Sage Publications, 2011).

[67] Jansen B, Geldof S, De Meester L, Orsini L (2011). Isolation and characterization of microsatellite markers in the waterflea Daphnia magna. Mol Ecol Resour.

[68] Orsini L, Spanier KI, De Meester L (2012). Genomic signature of natural and anthropogenic stress in wild populations of the waterflea Daphnia magna: validation in space, time and experimental evolution. Molecular Ecology.

[69] Development Core Team, R (eds Venables, W. N. & Smith, D. M.) (2008).

[70] Hessellund Andersen, B., Sørensen, J. & Miljøbevægelsen, N. Agriculture in Denmark. (The Nordic Council of Ministers, Denmark, 2015).

[71] Paul RJ (2004). Thermal acclimation in the microcrustacean Daphnia: a survey of behavioural, physiological and biochemical mechanisms. J Therm Biol.

[72] Winder M, Schindler DE (2004). Climate change uncouples trophic interactions in an aquatic ecosystem. Ecology.

[73] Wojtal-Frankiewicz A (2012). The effects of global warming on Daphnia spp. population dynamics: a review. Aquatic Ecology.

[74] Hansson LA (2013). Food-chain length alters community responses to global change in aquatic systems. Nature Climate Change.

[75] O’Neil JM, Davis TW, Burford MA, Gobler CJ (2012). The rise of harmful cyanobacteria blooms: The potential roles of eutrophication and climate change. Harmful Algae.

[76] Cambronero, C. M., Beasley, J., Kissane, S. & Orsini, L. Evolution of thermal tolerance in multifarious environments *Molecular Ecology* (in review). 10.1111/mec.14890 30298601

[77] De Meester L, Gomez A, Okamura B, Schwenk K (2002). The Monopolization Hypothesis and the dispersal-gene flow paradox in aquatic organisms. Acta Oecologica.

[78] Orsini L, Vanoverbeke J, Swillen I, Mergeay JDM (2013). L. Drivers of population genetic differentiation in the wild: isolation by dispersal limitation, isolation by adaptation and isolation by colonization. Molecular Ecology.

[79] Coors A, De Meester L (2008). Synergistic, antagonistic and additive effects of multiple stressors: predation threat, parasitism and pesticide exposure in Daphnia magna. Journal of Applied Ecology.

[80] Lopes Patricia C., Sucena Élio, Santos M. Emília, Magalhães Sara (2008). Rapid Experimental Evolution of Pesticide Resistance in C. elegans Entails No Costs and Affects the Mating System. PLoS ONE.

