# Supplementary material for: Predictability of the impact of multiple stressors on the keystone species Daphnia
Source: Sci Rep. 2018 Dec 4;8:17572. doi: 10.1038/s41598-018-35861-y (PMC6279757; doi:10.1038/s41598-018-35861-y)

Predictability of the impact of multiple stressors on the keystone species *Daphnia*

Maria Cuenca Cambronero[1], Hollie Marshall[1], Luc De Meester[2], Thomas Alexander Davidson[3], Andrew P. Beckerman[4#] and Luisa Orsini[1*#]

[1] Environmental Genomics Group, School of Biosciences, the University of Birmingham, Birmingham B15 2TT, UK

[2] Laboratory of Aquatic Ecology, Evolution and Conservation, University of Leuven, Ch. Deberiotstraat 32, 3000 Leuven, Belgium.

[3] Lake Group, Department of Bioscience, Aarhus University, Vejlsøvej 25, P.O. Box 314, DK-8600 Silkeborg, Denmark

[4]Department of Animal and Plant Sciences, University of Sheffield, Sheffield S10 2TN, U.K

[#]these authors contributed equally

Running head: Predictability of multiple stressors

Corresponding author:

Dr Luisa Orsini

Environmental Genomics Group, School of Biosciences, University of Birmingham, Birmingham B15 2TT, UK

Email: l.orsini@bham.ac.uk

Table S1. Environmental metadata

Paleolimnological and historical data are shown for the sedimentary archive of Lake Ring. For each radiometrically dated year, *Daphnia magna* ephippia count, Cladocera assemblage, carbamate insecticides, Loss on Ignition (LOI), transparency measured as Secchi disk depth, total Nitrates (TN) and total Phosphates (TP) are shown. Na = missing data. Data for transparency, TN and TP are available between 1971 and 1999.

Year	*D. magna* ephhipia	Cladocera assemblage	Carbaryl (T/year)	LOI	Average summer T (°C)	Secchi disk depth	TN	TP
2004	58	-	0.0	25.0	16.8	-	-	-
1999	94	0.2	5.9	26.7	16.2	2.5	0.8	0.1
1994	55	0.5	147.9	29.9	16.3	2.9	0.9	0.3
1987	28	0.4	104.7	31.2	15.6	2.1	1.7	0.7
1984	94	1.0	143.0	30.6	15.6	0.7	-	-
1977	16	0.4	28.9	33.4	15.5	0.9	1.7	1.8
1975	17	0.3	31.7	-	16.2	0.5	3.4	1.0
1971	19	0.5	14.5	33.3	16.3	0.7	1.7	1.3
1968	11	0.3	18.9	-	15.0	-	-	-
1964	7	-0.1	1.4	34.7	15.9	-	-	-
1959	13	-0.1	0.0	29.4	16.2	-	-	-
1942	31	-0.4	0.0	25.5	16.1	-	-	-
1931	22	-0.8	0.0	-	14.9	-	-	-
1925	22	-0.9	0.0	28.1	15.4	-	-	-
1918	3	-0.8	0.0	32.4	15.4	-	-	-
1908	2	-0.4	0.0	29.4	15.2	-	-	-
1900	1	0.1	0.0	-	16.2	-	-	-
1896	1	0.0	0.0	30.6	15.3	-	-	-

Table S2. Insecticides sold in Denmark

List of insecticides sold in Denmark according to the Danish county authority between 1955 and 2010 (www.middeldatabasen.dk). The total amount of each insecticide is show in tons. Yellow lines highlight carbamate insecticides, of which the commercial brand Carbaryl was used in this study.

	Pesticide	Tons
1	parathion	5272232
2	DNOC	2115794
3	TOTAL CARBAMATES	1660104
4	dimethoat	1538096
5	maleinhydrazid	1420773
6	methylbromid	1098882
7	cyanazin	850392
8	cupricarbonat-basisk	804526
9	pirimicarb	785775
10	mechlorprop-P	630959
11	malathion	587854
12	DDT	529960
13	allylalkohol	435780
14	carbamat, natrium-N-dimethyldithio	400275
15	cuprinaphthenat	350009
16	oxydemethon-methyl	320645
17	1,3-dichlorpropylen	306745
18	alloxydim-natrium	278147
19	2-bromo-2-nitropropan-1,3-diol	264741
20	monuron	207448
21	cypermethrin	198484
22	aluminiumphosphid	185896
23	fenitrothion	184380
24	carbofuran	174889
25	chlorpicrin	174876
26	imidacloprid	152108
27	4,5,2',4',6'-pentachlor-2-(chlormethylsulfonamid)-diphenylether-Na	149035
28	1,2-dichlorpropan	141230
29	methoxychlor	123448
30	formothion	112545
31	dinatrium-octaborat-tetrahydrat	110639
32	permethrin	99933
33	endosulfan	98880
34	carbaryl	96496
35	2,3,6-TBA	91776
36	isofenphos	90386

37	tau-fluvalinat	89793
38	furathiocarb	85299
39	thiometon	84823
40	diazinon	83406
41	azinphos-methyl	81389
42	kviksølv (bejdsemiddel)	80741
43	mercaptodimethur	63565
44	dichlorvos	62899
45	3-iodo-2-propynylbutyl-carbamat	58133
46	dinatrium-octaborat-tetrahydrat(natriumborat)	57322
47	parathion-methyl	54535
48	phosphamidon	54024
49	pyrethrin-IogII	54023
50	fenvalerat	51546
51	esfenvalerat	39646
52	trichlorfon	36733
53	dicofol	35623
54	phoxim	34775
55	metaldehyd	32936
56	cypermethrin, -alpha	31871
57	2,3-dihydro-6-methyl-5-phenylcarbamoyl-1,4-oxathiin	31489
58	alpha-cypermethrin	30372
59	dinatrium-tetraborat-decahydrat(natriumborat)	30301
60	methoxyethylmercurisilikat	29634
61	thiourinstof	29256
62	furmecyclox	28596
63	aldicarb	27748
64	cuprihexafluorsilikat	26732
65	chlordioxid	26353
66	phosalon	25409
67	dieldrin	25015
68	chlorfenvinphos	24706
69	ronnel	21358
70	acephat	20962
71	N,N-diethyl-m-toluamid	20962
72	mevinphos	19538
73	lambda-cyhalothrin	17636
74	2,2-dibrom-2-cyanoacetamid	16642
75	diflubenzuron	16408
76	chlorpyrifos	16263
77	bromophos	15676
78	deltamethrin	15396

79	trichloronat	15060
80	cyromazin	14788
81	nicotin	14466
82	jodfenphos	14357
83	icaridin	13750
84	propoxur	12957
85	thallium(I)sulfat	12302
86	d-trans-allethrin	11786
87	cuprihydroxicarbonat	11418
88	tetrasul	11290
89	dinatrium-octaborat(natriumborat)	11075
90	tetradifon	10871
91	Amitraz	10721
92	hydroxy isobutyl piperidin carboxylat	9718
93	chlorfenson	9073
94	2-(thiocyanomethylthio)-benzothiazol	8665
95	azamethiphos	8549
96	dienochlor	8396
97	chlorsulfuron	7577
98	dibrom	7506
99	rotenon	7395
100	cyhexatin	7114
101	sulfotep	7082
102	oxamyl	6317
103	methomyl	5816
104	etrimphos	5558
105	methidathion	5236
106	betacyfluthrin	4807
107	chlorphenamidin	4799
108	cuprioctanoat	4711
109	2,4-dinitrophenol	4389
110	dimethylphthalat	4070
111	ethiofencarb	3985
112	tetrachlorvinphos	3829
113	thiamethoxam	3748
114	hjortetakolie	3642
115	bioresmethrin	3608
116	citronellol	3518
117	phosmet	3390
118	p-menthan-3,8-diol	3069
119	thiophanox	2951
120	tecnazen	2902

121	fenbutatin-oxid	2719
122	amidosulfuron	2698
123	warfarin	2305
124	2-methyl-4-isothiazolin-3-on	2203
125	propargit	2148
126	clofentezin	1869
127	tefluthrin	1768
128	chloralose (alpha)	1553
129	fenpropathrin	1488
130	2-ethylhexan-1,3-diol	1274
131	propetamphos	1188
132	fenazaquin	924
133	calciumpolysulfid	922
134	methopren	691
135	spinosad	616
136	indoxacarb	180

Table S3. Genotypes used in the experiments.

Individual ID, population ID and age are shown for the genotypes used in the experiments. All genotypes were used in the common garden experiments. The genotypes with an asterisk (*) were used in the competition experiment The multilocus microsatellite profile for the genotypes is available at https://doi.org/10.5061/dryad.5k6t6.

Individual ID	Population ID	age
LRV 0_1*	CWP	>1999
LRV 0.5_3	CWP	>1999
LRV 2_1*	CWP	>1999
LRV 2.5_9*	CWP	>1999
LRV 2.5_11*	CWP	>1999
LRV 3_4	CWP	>1999
LRV 3.5_1*	CWP	>1999
LRV 3.5_2	CWP	>1999
LRV 3.5_15*	CWP	>1999
LRV 3_6	CWP	>1999
LRV 6_2*	PP	1980-1990
LRV 6_3	PP	1980-1990
LRV 6.5_5	PP	1980-1990
LRV 6.5_7	PP	1980-1990
LRV 7_3	PP	1980-1990
LRV 7_5	PP	1980-1990
LRV 7.5_4*	PP	1980-1990
LRV 8.5_3*	PP	1980-1990
LRV 8_7	PP	1980-1990
LRV 9.5_1*	PP	1980-1990
LRV 9.5_3*	PP	1980-1990
LRV 9_6*	PP	1980-1990
LRV 9_20*	PP	1980-1990
LRV 12_2	EP	1960-1970
LRV 12_3*	EP	1960-1970
LRV 12_4	EP	1960-1970
LRV 12.5_1	EP	1960-1970
LRV 13_1*	EP	1960-1970
LRV 13_2*	EP	1960-1970
LRV 13_3*	EP	1960-1970
LRV 13.5_1*	EP	1960-1970
LRV 14.5_1*	EP	1960-1970
LRV 15.5_1*	EP	1960-1970

Figure S1. ***Daphnia magna*** **hatching success**

Percentage of *D. magna* dormant embryos that successfully hatched in the laboratory across the sedimentary archive. This figure is modified from Cambronero and Orsini (2018).

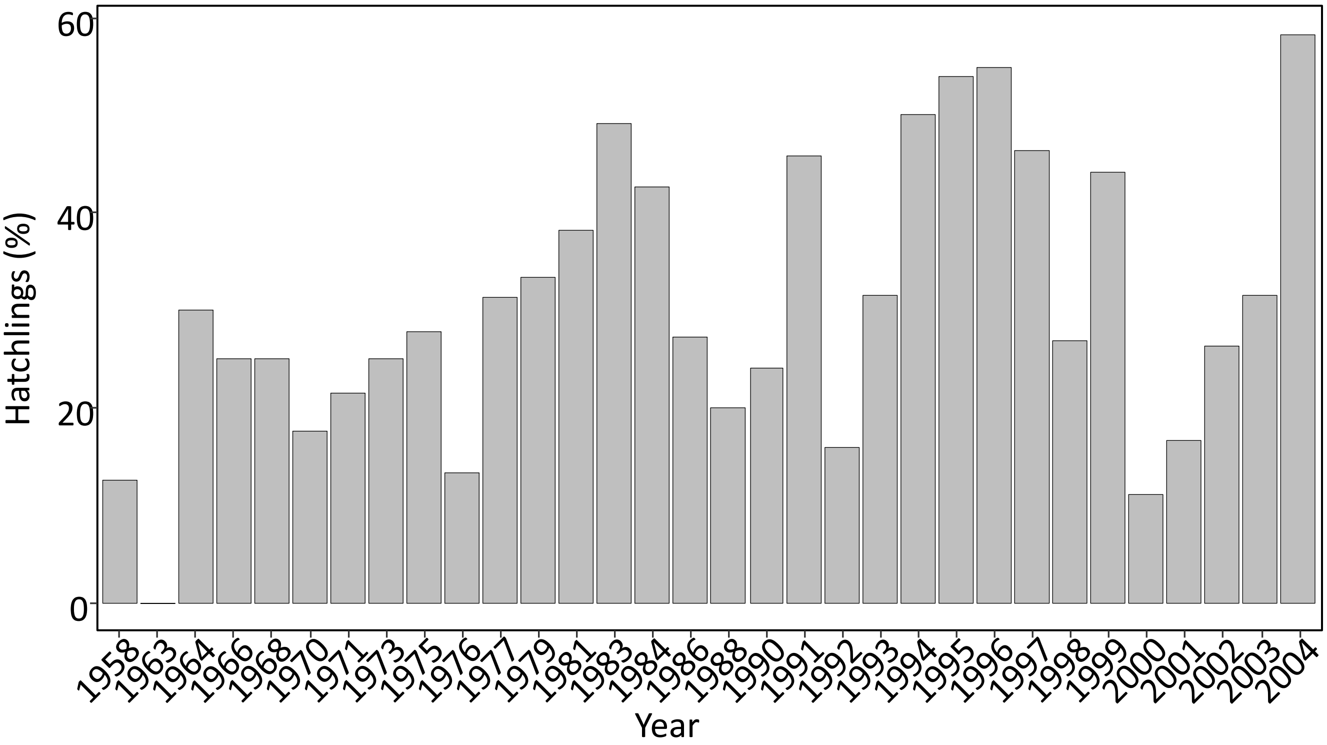

Figure S2. Mortality

Mortality per population and experiment: CGE1 (Temperature), CGE2 (Temperature and Food) and CGE3 (Temperature and Carbaryl) measured over 21 days. For CGE2 high (continuous lines) and low (dotted lines) concentrations of food are shown. For CGE3 high (continuous lines) and low (dotted lines) concentrations of Carbaryl are shown. The concentrations are as in Figure 1. Mortality was calculated with survival analysis in R using the rms package (https://cran.r-project.org/web/packages/rms/rms.pdf).

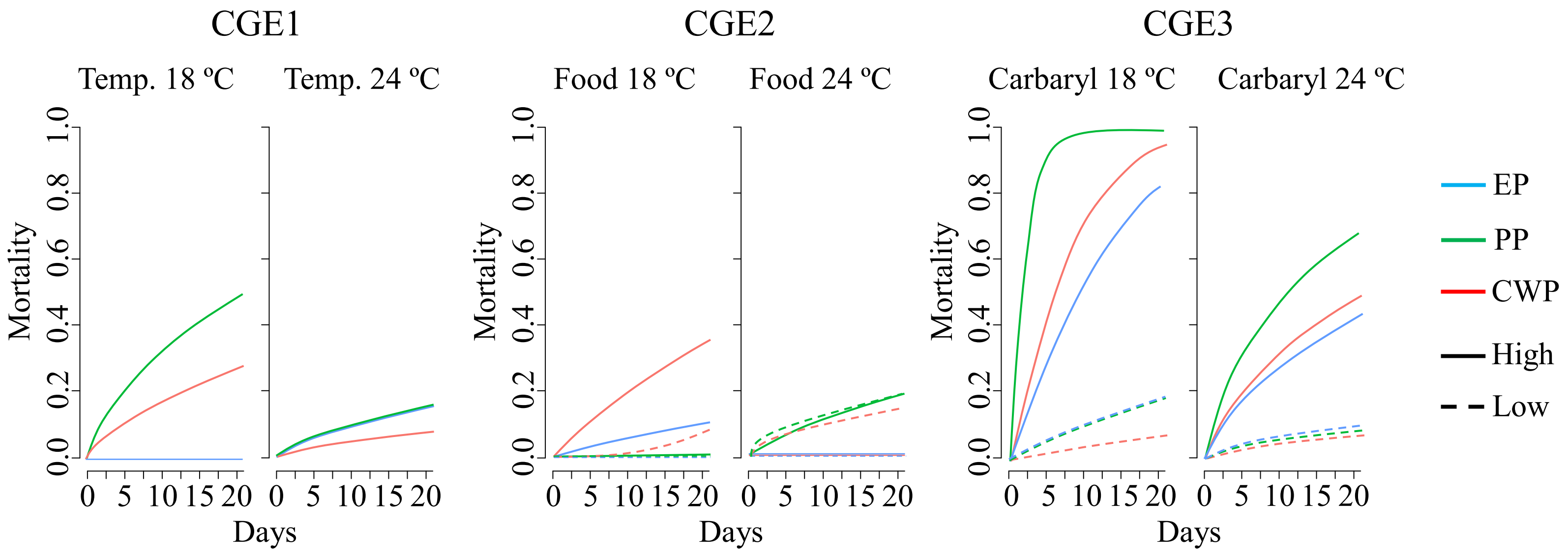

Supplement: Supplementary file 1 — Supplementary Information [file 41598_2018_35861_MOESM1_ESM.pdf]
